# Relationships between hand grip strength and gait parameters measured using a foot-mounted sensor in non-laboratory settings in older women

**DOI:** 10.1038/s41598-025-14442-w

**Published:** 2025-08-11

**Authors:** Takuma Inai, Tomoya Takabayashi

**Affiliations:** 1 Integrated Research Center for Self-Care Technology, National Institute of Advanced Industrial Science and Technology (AIST), 2217-14 Hayashi-cho, Takamatsu, Kagawa 761-0395 Japan; 2 Health and Medical Research Institute, National Institute of Advanced Industrial Science and Technology (AIST), Takamatsu, Kagawa Japan; 3 Research Institute on Human and Societal Augmentation, National Institute of Advanced Industrial Science and Technology (AIST), Kashiwa, Chiba, Japan; 4 Institute for Human Movement and Medical Sciences, Niigata University of Health and Welfare, Niigata, Niigata Japan

**Keywords:** Gait, Older women, Wearable sensor, Foot, Hand grip strength, Geriatrics, Health policy

## Abstract

Muscle weakness is a significant concern in older women, as it increases the risk of falls and fractures. To develop practical tools for early detection, it is important to understand how hand grip strength relates to gait parameters in non-laboratory settings. This study examined the relationship between hand grip strength and gait characteristics in older women, using a foot-mounted sensor during natural walking. Publicly available data from 55 women aged > 70 years were analyzed. Each woman wore an inertial measurement unit attached to their shoe and completed a 30-min walking task. Hand grip strength was used as a general indicator of muscle strength. The mean and coefficient of variation (CV) were calculated for 18 gait parameters, including spatiotemporal parameters and foot pitch angles. Hand grip strength showed significant correlations with several mean gait parameters, including the percentage of the stance phase (*r* = −0.57) and the timing of the minimum foot pitch angle (*r* = −0.59). While hand grip strength was significantly correlated with the CV of several gait parameters, the strongest *r* value was −0.34. These findings suggest that mean gait parameters may better reflect hand grip strength than their CV.

**Supplementary Information:**

The online version contains supplementary material available at 10.1038/s41598-025-14442-w.

## Introduction

Muscle weakness in older adults is a significant concern because it impairs balance^1–3^ and substantially increases the risk of falls^4–6^. These falls often lead to fractures such as distal radius^7,8,^, lumbar compression^8, ^and femoral neck fractures^7–11^ that can greatly reduce the ability to perform daily activities^12–14^. Muscle weakness tends to develop earlier in women than in men, likely due to menopause^15^. Additionally, women face higher rates of osteoporosis^16^, sarcopenia^17,18^, and fracture risk^16^ compared to men. For these reasons, early assessment of muscle strength is critical for timely intervention and effective fall prevention, particularly in older women.

Several devices are currently available to assess muscle strength, including the Biodex system^19^ and hand-held dynamometers^20,21^. However, the Biodex system is expensive and impractical for regular or personal use. While hand-held dynamometers are more affordable, they require individuals to exert maximum voluntary effort, which may be difficult for some older women to sustain consistently. These challenges highlight the need for alternative ways to assess muscle strength that leverage data gathered during everyday activities.

Inertial measurement units (IMUs) are inexpensive, lightweight, compact, and easy to use in everyday life. Collecting gait data while wearing shoes equipped with IMUs offers a significant practical advantage^22,23^. Although previous studies have reported associations between muscle strength and gait parameters^24–39^, no studies have examined these relationships using foot-mounted sensors in non-laboratory settings. Moreover, gait characteristics are known to differ between laboratory and non-laboratory settings^40,41^, making it important to explore these associations in non-laboratory settings. Understanding these connections could help assess muscle strength through gait analysis during normal daily walking.

This study aimed to investigate the relationship between hand grip strength and gait parameters in older women using a foot-mounted sensor in non-laboratory settings. Hand grip strength was used as an indicator of muscle strength in this study, as it is strongly correlated with lower limb strength^42^ and is widely used in diagnosing sarcopenia^43^.

## Methods

### Database and data collection

We used the GSTRIDE database, previously described in a published study^44^ and publicly available in Zenodo^45^. A brief overview of the dataset is provided here for reference^44^. The study included 163 adults aged > 70 years (45 men, 118 women). The original study assessed fall history over the last year, along with a range of anatomical, functional, and cognitive variables, including body mass, height, body mass index (BMI), and the Global Deterioration Scale (GDS). Additional assessments included the 4-meter walk test (duration and gait speed); frailty indicators such as hand grip strength (measured using an analog hydraulic hand dynamometer [JAMAR Dynamometer, Talexco, Spain] and a digital dynamometer [MAP 80K1S Dynamometer, KERN & SOHN, Germany])^44^; the Short Physical Performance Battery; the Timed Up and Go test; the Short Falls Efficacy Scale–International; and gait data collected via IMUs. For gait data collection, an IMU was attached to the top of the participant’s foot (left or right) using an elastic strap. The coordinate system was defined as follows: x axis (medial/lateral), y axis (anterior/posterior), and z axis (superior/inferior). The participants first stood still for 30 s, then began walking in a non-laboratory setting (i.e., indoors or outdoors). Acceleration and angular velocity were recorded throughout a 30-min walking session. Two types of IMUs were used: LSM6DSRX (STMicroelectronics, CH) and Physilog 6 S (GaitUp, CH)^44^. Although both types were included in the dataset, a previous study^46^ found that differences between the devices had minimal impact on gait parameter estimates, with mean relative errors ranging from 2.26 to 5.04%. The study^44^ was approved by the Ethics Committee for Research with Medicines of Hospital Universitario La Paz (Ref. HULP: PI-4486) and conducted within the framework of the Analysis of the Gait Pattern through the Design of an Electronic Prototype and a Monitoring App project (G-STRIDE, Ref. M2451). All methods were performed in accordance with the relevant guidelines and regulations. All participants or their relatives provided written informed consent before participation.

### Data analysis

The sample size was determined using R version 4.3.0 (R Development Core Team). Correlation analysis was performed to investigate the relationship between hand grip strength and gait parameters. An a priori power analysis was conducted using the following parameters: an effect size (Cohen’s *r*) of 0.37, a significance level (α) of 0.05, and statistical power (1–β) of 0.80. The effect size was based on a previous study^47^, which reported a correlation coefficient of − 0.37 between gait velocity and the Standardized Frailty Criteria. We used the pwr.r.test function from the pwr package to calculate the required sample size (see Supplementary material for details). The sample size required for this study was determined to be 54.

**Fig. 1:**
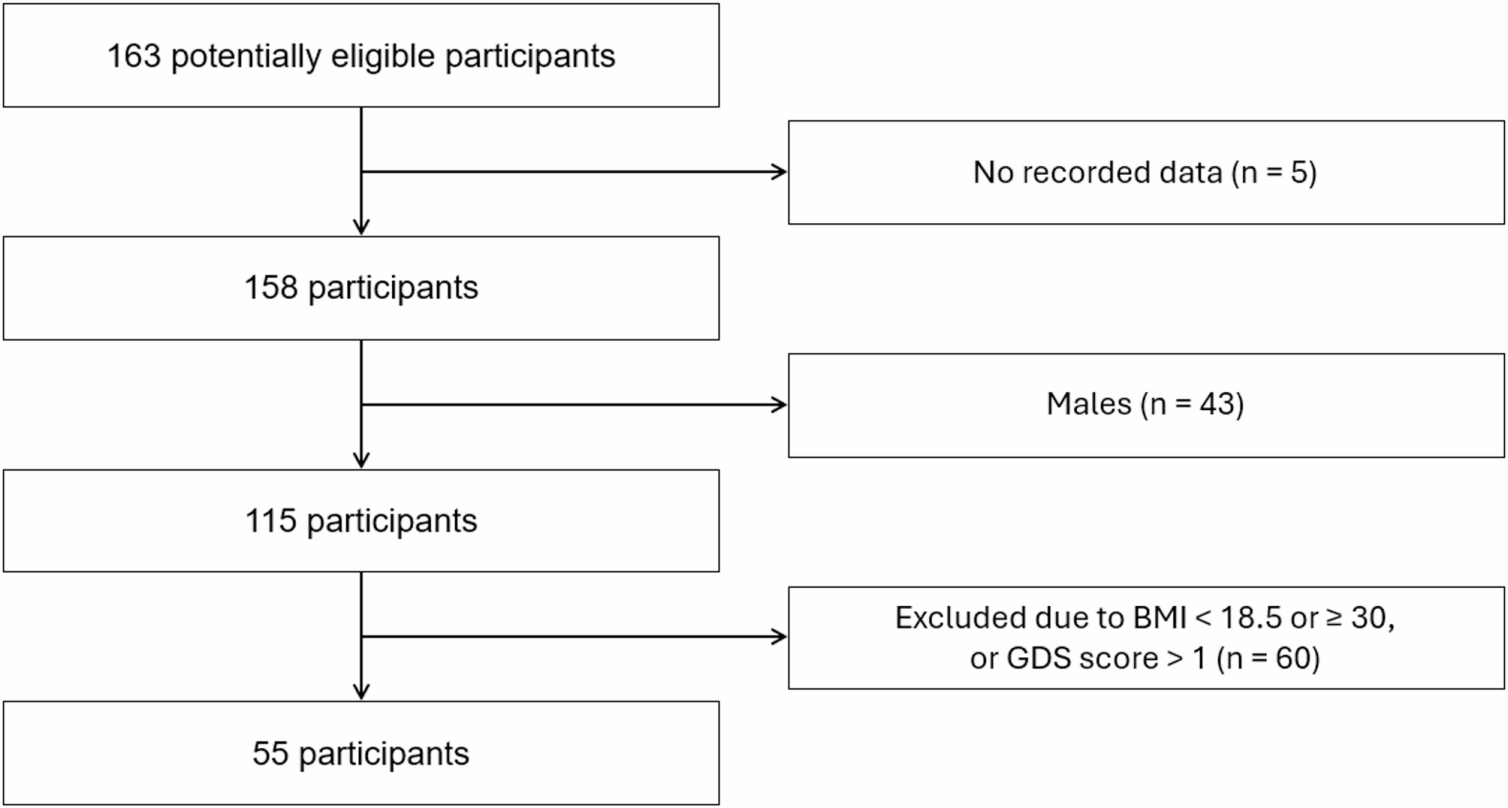
Flowchart of participant inclusion and exclusion criteria. Analysis of 23 older women in the normal hand grip strength group and 32 older women in the low hand grip strength group.

Of the 163 participants, gait data from 55 were included in the analysis based on the criteria outlined below (Figure 1; Table 1). The inclusion criterion was female sex. A previous study^48^ reported that women have lower muscle strength than men, and other studies^16,49^ have also found a higher prevalence of osteoporosis and increased risk of fractures in women compared to men. Therefore, the present study focused on women. The exclusion criteria were as follows: (1) GDS score > 1 and (2) BMI > 30 kg/m². Cognitive function^50–52^ and BMI^53^ may influence gait parameters. Therefore, these exclusion criteria were applied to minimize potential confounding factors.

**Table 1:** Demographics of participants.

	Mean	(SD)
Age	70–74 75–79 80–84 85–89 90–94 95–99	[*n* = 7] [*n* = 16] [*n* = 15] [*n* = 10] [*n* = 6] [*n* = 1]
Height, m	1.57	(0.10)
Body mass, kg	60.8	(8.7)
Body mass Index	24.7	(3.0)
Hand grip strength, N/kg	0.28	(0.10)

**Fig. 2:**
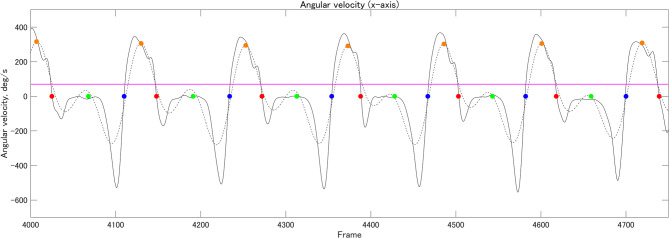
Gait event detection. Red, green, blue, and orange circles indicate heel contact, foot flat, toe-off, and local maximum value (threshold: ≥70 deg/s), respectively.

Calibrated IMU data were provided in the TXT format^45^. We developed custom scripts to calculate all the Gait Parameters using MATLAB version R2024b (MathWorks) (see Gait Parameters section for further details). First, the IMU data (acceleration and angular velocity) were imported and filtered using a fourth-order Butterworth filter with a zero-phase lag and a cutoff frequency of 10 Hz^54^. Given its high reliability in detecting gait events, the zero-crossing method^55^ was used to identify heel contact and toe-off based on angular velocity along the x-axis (Figure 2). Foot flat was defined as the midpoint between heel contact and toe-off^56^ (Figure 2).

The IMU orientation was estimated using a Kalman filter algorithm. Gravitational acceleration was calculated from 15 s of standing data and used to remove the gravitational component along the z-axis of the global coordinate system (GCS). The IMU velocity was obtained by integrating the acceleration data between two consecutive ipsilateral foot-flat events. A zero-velocity update was applied to correct velocity estimates^57^. The IMU trajectory was then computed by integrating the corrected velocity. It was assumed that the IMU’s height (Z = 0) remained constant between the foot flat and the subsequent ipsilateral foot flat events. Based on this assumption, drift along the z-axis was corrected using the same method described previously^57^. Finally, the trajectory was rotated so that the Y-axis aligned with the vector connecting the IMU positions at the two foot-flat events.

Several outliers were identified in the gait parameters of the dataset. To enhance the robustness of our analysis, we removed these outliers based on predefined criteria. Specifically, we calculated the mean and standard deviation (SD) of stride time for each participant across multiple trials. Gait trials were excluded if their stride times fell outside the range of mean ± 2 SD. Additionally, trials were excluded if the maximum foot pitch angle exceeded 50 degrees, or the minimum foot pitch angle was below −80 degrees. Fifty gait cycles without outliers were included for analysis for each participant.

### Gait parameters

**Fig. 3:**
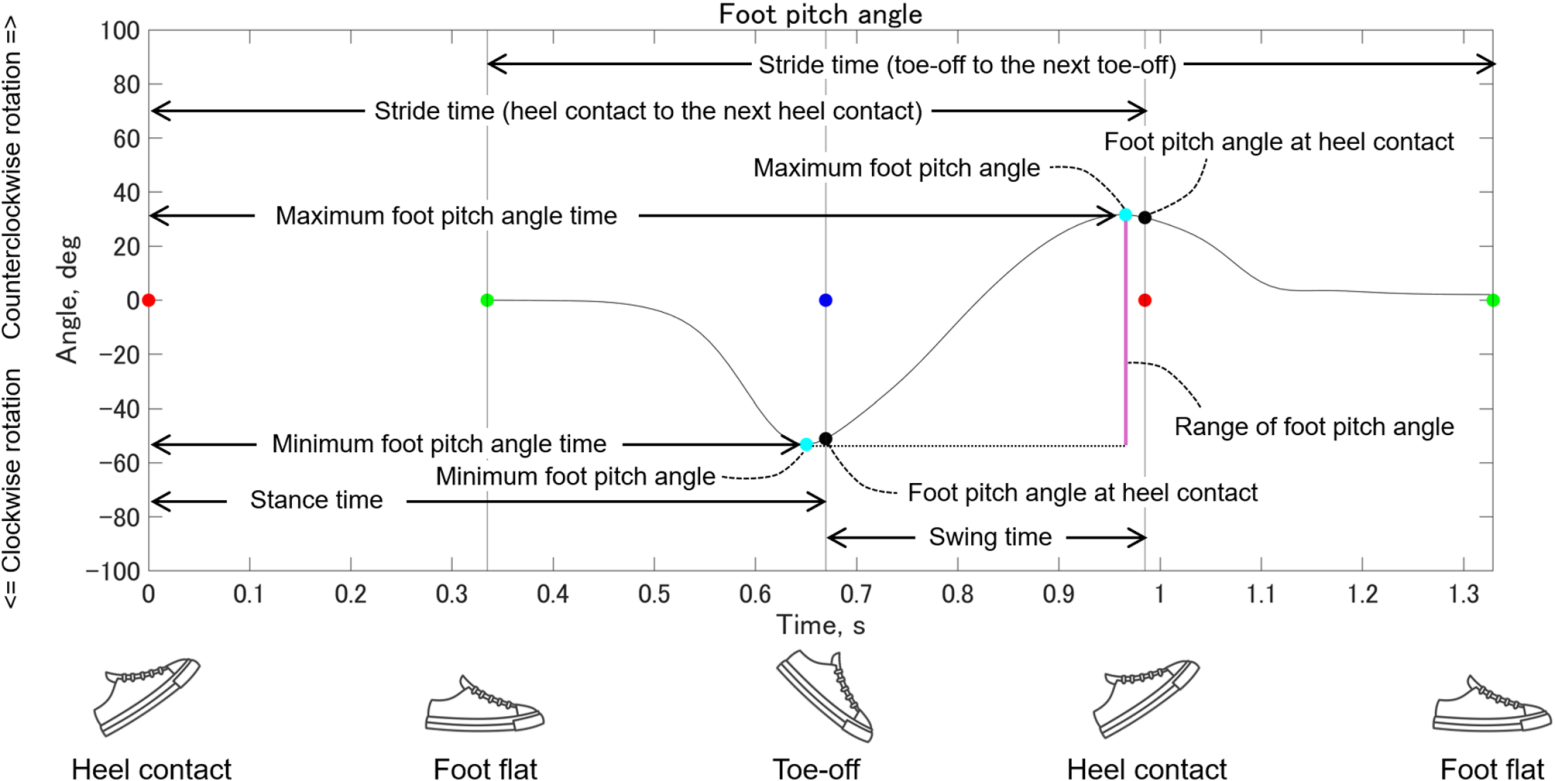
Spatial gait parameters and foot pitch angles. In the sagittal plane, when viewed from the right side of the foot, counterclockwise foot rotation (dorsiflexion) is represented by positive values, and clockwise rotation (plantarflexion) by negative values. A foot orientation parallel to the ground corresponds to 0 degrees. The red, green, and blue circles indicate heel contact, foot flat, and toe-off, respectively.

Muscle weakness may influence both the mean and variability of gait parameters^22,23,31,58^. Therefore, this study focused on mean values and coefficient of variation (CV). Figure 3 shows details of spatial gait parameters and foot pitch angles. Gait speed was calculated as the ratio of stride length to stride time (toe-off to the next toe-off). Stride length normalized to body height, was defined as the distance between the foot flat and the subsequent ipsilateral foot flat position. Cadence was calculated using stride time (heel contact to the next heel contact), which was defined as the interval between two consecutive heel contacts on the same side. Stance time was defined as the duration from heel contact to toe-off, whereas swing time was defined as the duration from toe-off to the next heel contact. The percentage of the stance phase was calculated by dividing stance time by stride time (heel contact to the next heel contact). Since stance and swing phase percentages are linearly dependent, only the percentage of the stance phase was analyzed. Foot pitch angle was computed in the sagittal plane using the Z-axis of the GCS and the local coordinate system of the foot. Pitch angles at heel contact and toe-off, as well as the minimum and maximum pitch angles during the stride, were extracted. The range of foot pitch angles was calculated as the difference between the minimum and maximum angles. The minimum and maximum foot pitch angle times were defined as the durations from heel contact to their respective occurrence within the stride. These times were then normalized to stride time (heel contact to the next heel contact) and expressed as percentages of stride duration (i.e., [minimum or maximum foot pitch angle time/stride time] × 100). The walk ratio was calculated as step length (half of the stride length) divided by cadence. Step speed was defined as the average foot velocity during the swing phase.

### Muscle strength

Handgrip strength is widely used in the diagnosis of sarcopenia^43^, as it reflects overall muscle strength. In this study, it was used as an index of muscle strength. The database provided the maximum hand grip strength value for each participant⁴⁴ (rather than a mean value). Handgrip strength was measured using both an analog hydraulic hand dynamometer (JAMAR Dynamometer, Talexco, Spain) and a digital dynamometer (MAP 80K1S Dynamometer, KERN & SOHN, Germany)^44^.

### Statistical analysis

The normality of each variable was assessed using the Shapiro-Wilk test. Based on the results, either Pearson or Spearman correlation coefficients were used to assess the relationships between variables. Statistical significance was set at *p* < 0.05. Based on a previous study^59^, effect sizes (Cohen’s *r*) were calculated and defined as negligible (Cohen’s *r* < 0.1), small (0.1 ≤ Cohen’s *r* < 0.3), medium (0.3 ≤ Cohen’s *r* < 0.5), and large (Cohen’s *r* ≥ 0.5). All statistical analyses were performed using R.

## Results

Table 2 shows the relationship between mean gait parameters and hand grip strength. Significant positive correlations were observed between gait speed, stride length, cadence, foot pitch angle at heel contact, maximum foot pitch angle, range of foot pitch angle, and foot speed during the swing phase. In contrast, significant negative correlations were observed for stride time, stance time, percentage of stance phase, minimum foot pitch angle, minimum foot pitch angle time, maximum foot pitch angle time, and timing of the minimum foot pitch angle.

**Table 2:** Correlations between mean gait parameters and hand grip strength.

	Mean	(SD)	*p*-value	*r*	Effect size	Interpretation	
Gait speed, m/s	0.88	(0.32)	**0.0001**	0.49	0.49	Medium	a
Stride length, m/HT	0.64	(0.14)	**0.0020**	0.41	0.41	Medium	b
Cadence, steps/min	102.3	(16.0)	**0.0006**	0.45	0.45	Medium	a
Stride time, sec	1.23	(0.29)	**0.0006**	−0.45	0.45	Medium	b
Stance time, sec	0.89	(0.24)	**0.0002**	−0.48	0.48	Medium	b
Swing time, sec	0.34	(0.06)	0.1922	−0.18	0.18	Small	b
Percentage of stance phase, %	71.3	(3.1)	**<0.0001**	−0.57	0.57	Large	b
Foot pitch angle at toe-off, deg	−46.9	(9.2)	0.0624	−0.25	0.25	Small	a
Foot pitch angle at heel contact, deg	18.5	(7.7)	**0.0179**	0.32	0.32	Medium	a
Minimum foot pitch angle, deg	−48.8	(9.6)	**0.0371**	−0.28	0.28	Small	a
Maximum foot pitch angle, deg	19.7	(7.7)	**0.0127**	0.33	0.33	Medium	a
Range of foot pitch angle, deg	68.5	(15.3)	**0.0103**	0.34	0.34	Medium	a
Minimum foot pitch angle time, sec	0.86	(0.25)	**0.0001**	−0.49	0.49	Medium	b
Maximum foot pitch angle time, sec	1.19	(0.25)	**0.0008**	−0.44	0.44	Medium	b
Timing of minimum foot pitch angle, %	69.3	(3.4)	**<0.0001**	−0.59	0.59	Large	b
Timing of maximum foot pitch angle, %	97.3	(3.3)	0.5710	−0.08	0.08	Negligible	b
Walk ratio, mm/(steps/min)	5.1	(1.8)	0.0527	0.26	0.26	Small	b
Foot speed during the swing phase, m/s	2.52	(0.73)	**0.0005**	0.45	0.45	Medium	a

Bold values indicate *p* < 0.05. HT: Height. a: Pearson’s test. b: Spearman’s test.

Table 3 shows the relationships between the CV of gait parameters and hand grip strength. Significant negative correlations were found between gait speed, cadence, stride time, stance time, swing time, percentage of stance phase, minimum foot pitch angle, range of foot pitch angle, minimum foot pitch angle time, maximum foot pitch angle time, timing of minimum foot pitch angle, walk ratio, and foot speed during the swing phase.

**Table 3:** Correlations between the coefficients of variation of gait parameters and hand grip strength.

	Mean	(SD)	*p*-value	*r*	Effect size	Interpretation	
Gait speed, %	12.5	(12.5)	**0.0164**	−0.32	0.32	Medium	b
Stride length, %	13.0	(25.2)	0.0559	−0.26	0.26	Small	b
Cadence, %	6.5	(6.9)	**0.0297**	−0.29	0.29	Small	b
Stride time, %	9.8	(14.5)	**0.0178**	−0.32	0.32	Medium	b
Stance time, %	12.4	(19.4)	**0.0222**	−0.31	0.31	Medium	b
Swing time, %	7.7	(7.0)	**0.0111**	−0.34	0.34	Medium	b
Percentage of stance phase, %	3.0	(3.6)	**0.0305**	−0.29	0.29	Small	b
Foot pitch angle at toe-off, %	7.8	(3.9)	0.0626	−0.25	0.25	Small	b
Foot pitch angle at heel contact, %	26.4	(43.2)	0.0558	−0.26	0.26	Small	b
Minimum foot pitch angle, %	7.6	(4.0)	**0.0496**	−0.27	0.27	Small	b
Maximum foot pitch angle, %	19.1	(11.9)	0.0610	−0.25	0.25	Small	b
Range of foot pitch angle, %	8.1	(4.3)	**0.0315**	−0.29	0.29	Small	b
Minimum foot pitch angle time, %	12.7	(19.8)	**0.0174**	−0.32	0.32	Medium	b
Maximum foot pitch angle time, %	9.9	(15.2)	**0.0157**	−0.32	0.32	Medium	b
Timing of minimum foot pitch angle, %	3.1	(3.8)	**0.0412**	−0.28	0.28	Small	b
Timing of maximum foot pitch angle, %	2.1	(4.6)	0.7656	0.04	0.04	Negligible	b
Walk ratio, %	22.2	(61.5)	**0.0439**	−0.27	0.27	Small	b
Foot speed during the swing phase, %	9.3	(6.9)	**0.0373**	−0.28	0.28	Small	b

Bold values indicate *p* < 0.05. a: Pearson’s test. b: Spearman’s test.

## Discussion

This study examined the relationship between hand grip strength and gait parameters in older women using a foot-mounted sensor in non-laboratory settings. The key findings revealed significant correlations between hand grip strength and several mean gait parameters, including gait speed (*r* = 0.49 [medium]), percentage of the stance phase (*r* = −0.57 [large]), and timing of the minimum foot pitch angle (*r* = −0.59 [large]), as shown in Table 2. Our results suggest that a walking pattern associated with lower hand grip strength is characterized by reduced gait speed, resulting from shorter stride length and lower cadence, as well as an increased percentage of the stance phase. Although previous studies have reported associations between muscle strength and gait parameters^24–39^, only a few have used shoe-mounted IMUs to investigate these relationships^29,31^. Furthermore, all existing studies conducted their walking tests in controlled laboratory environments. Given that gait parameters can differ between laboratory and non-laboratory settings^40,41^, it is important to examine these associations in non-laboratory settings. To the best of our knowledge, this is the first study to investigate the relationship between hand grip strength and gait parameters in older women using foot-mounted sensors in non-laboratory settings. These findings may help support the development of practical methods for assessing muscle strength using gait data collected during natural walking in non-laboratory settings.

The present study demonstrated a positive association between hand grip strength and mean gait speed with a medium effect size (*r* = 0.49). This finding is consistent with a previous simulation study^60^ reporting that muscle weakness leads to reduced gait speed, and with an experimental study^25^ reporting a similar association. Although, in our study, gait speed measured using foot-mounted sensors in non-laboratory settings was estimated through numerical integration of acceleration data, our results suggest that gait speed can serve as a useful indicator for assessing hand grip strength in older women.

In the present study, reduced hand grip strength was negatively associated with stance time (*r* = −0.48 [medium]), the percentage of the stance phase (*r* = −0.57 [large]), and the timing of the minimum foot pitch angle (*r* = −0.59 [large]). A previous study^31^ assessed gait parameters using a gait analysis system that incorporated shoe-type data loggers with embedded IMUs on both outsoles during a straight 19-meter overground walk. The researchers examined the relationships between gait parameters and hand grip strength and reported a negative association between hand grip strength and stance phase duration during preferred-speed walking (*r* = −0.233). Therefore, previous findings regarding stance phase duration are consistent with our results. However, the previous study^31^ did not evaluate the percentage of the stance phase or the timing of the minimum foot pitch angle. Since these two variables demonstrated stronger associations with hand grip strength than stance time in our analysis, we suggest that they may serve as more sensitive indicators for assessing muscle strength in older adults.

Previous studies have indicated that increased variability in gait parameters correlates with higher fall risks^61–64^ and muscle weakness^31^. Therefore, we investigated the relationship between hand grip strength and the mean gait parameters and their CVs. Significant associations were found between hand grip strength and several CVs of gait parameters, including gait speed (*r* = −0.32 [medium]) and swing time (*r* = −0.34 [medium]), as detailed in Table 3. While hand grip strength showed significant correlation with the CVs of several gait parameters, the strongest correlation observed was *r* = −0.34 (swing time). The effect size for CVs and mean gait parameters ranged from 0.27 to 0.34 (small to medium) and 0.28 to 0.59 (small to large), respectively. A previous study^65^ has indicated that gait variability is also influenced by cognitive function. However, the present study only included older women without cognitive decline (i.e., GDS = 1). Notably, if older women with cognitive decline were analyzed, different results may be obtained. Since effect sizes (e.g., small, medium, and large) reflect the strength of the relationship between hand grip strength and gait parameters measured using a foot-mounted sensor, our findings suggest that the CVs of gait parameters might be insignificant. In contrast, the mean values of gait parameters may be more important than their CVs for older women without cognitive decline when assessing hand grip strength.

We also conducted additional analyses that retained outliers, recognizing that these data points may offer valuable insights in a community-based sample. However, the correlation coefficients did not change significantly (Supplementary Tables S1 and S2). Specifically, hand grip strength remained significantly associated with gait speed (*r* = 0.49 [medium] in Table 2 vs. *r* = 0.48 [medium] in Supplementary Table S1), percentage of the stance phase (*r* = −0.57 [large] vs. *r* = −0.51 [large]), and timing of the minimum foot pitch angle (*r* = −0.59 [large] vs. *r* = −0.48 [medium]). Although the impact of outlier removal was minimal, excluding outliers may still be beneficial for improving accuracy and interpretability.

A systematic review^66^ noted that most studies using IMUs during walking have placed the sensors on the lower back, due to its proximity to the body’s center of mass. One such study^67^ reported that gait variability measured from a lower-back IMU was associated with muscle strength. However, wearing a device in that location can interfere with daily activities, pressing against chairs when seated, striking the floor when lying down, or making it difficult to dress and undress. These practical limitations may reduce compliance in non-laboratory settings. In contrast, shoe-mounted IMUs offer a more user-friendly alternative, as they require no behavioral changes, participants simply wear their shoes as usual. A previous study using the same dataset⁴⁷ found that this placement was comfortable and did not affect walking behavior. Additionally, a systematic review^55^ reported that foot-based algorithms outperform trunk-based ones in detecting gait events. Thus, foot-mounted IMUs enable accurate gait analysis while offering superior practicality for daily use.

The Asian Working Group for Sarcopenia (2019) recommends using hand grip strength as a standard measure of muscle strength[^43^. Furthermore, previous research⁴² has shown strong correlations between grip strength and lower limb strength, including hip, knee, and ankle muscle groups. Based on this evidence, we used hand grip strength as a proxy for overall muscle strength in this study. However, since grip strength does not directly reflect lower limb strength, results might differ if other measures (e.g., knee extension strength) were assessed. Still, our findings provide meaningful insight into the potential for assessing muscle strength from gait data collected during natural walking in non-laboratory settings.

This study has some limitations. First, gait data were obtained from 30-min walking sessions conducted in non-laboratory settings, which may not fully reflect typical daily walking patterns. Therefore, further investigation is required to confirm the generalizability of the findings to daily walking. Second, the study included older women only so the results may not be generalizable to middle-aged men and women or older men. Further research is needed to explore the relationship between hand grip strength and gait parameters measured using a foot-mounted IMU in these populations.

## Conclusion

This study investigated the relationships between hand grip strength and gait parameters in older women using a foot-mounted sensor in non-laboratory settings. These findings demonstrated significant associations between hand grip strength and both the mean values and CVs of the gait parameters. These results offer valuable insights into the potential for assessing muscle strength in older adults using gait data collected during natural walking in non-laboratory environments.

## Supplementary Information

Below is the link to the electronic supplementary material.

Supplementary Material 1

## Data Availability

The datasets analyzed during the current study are available at https://zenodo.org/records/8003441.
