# Supplementary material for: Relationships between hand grip strength and gait parameters measured using a foot-mounted sensor in non-laboratory settings in older women
Source: Sci Rep. 2025 Aug 11;15:29375. doi: 10.1038/s41598-025-14442-w (PMC12340058; doi:10.1038/s41598-025-14442-w)
Supplement: Supplementary file 1 — Supplementary Material 1 [file 41598_2025_14442_MOESM1_ESM.pdf]

Supplementary material

Power analysis using R

```
# R version 4.3.0
install.packages('pwr')
library(pwr)

# Parameters
n <- NULL
r <- 0.37
sig.level <- 0.05
power <- 0.8
alternative <- "two.sided"

# Calculation using pwr package
pwr.r.test(n,r,sig.level,power,alternative)

# Specific Calculation
r <- abs(r)
p.body <- quote({
  ttt <- qt(sig.level/2, df = n - 2, lower = FALSE)
  rc <- sqrt(ttt^2/(ttt^2 + n - 2))
  zr <- atanh(r) + r/(2 * (n - 1))
  zrc <- atanh(rc)
  pnorm((zr - zrc) * sqrt(n - 3)) + pnorm((-zr - zrc) * sqrt(n - 3))})

n <- uniroot(function(n) eval(p.body) - power, c(4 + 1e-10, 1e+09))$root

METHOD  <-  "approximate  correlation  power  calculation  (arctangh
transformation)"
structure(list(n = n, r = r, sig.level = sig.level, power = power,
              alternative  =  alternative,  method  =  METHOD),  class  =
"power.htest")
```

Execution results

```
>
> # Parameters
> n <- NULL
> r <- 0.37
> sig.level <- 0.05
> power <- 0.8
> alternative <- "two.sided"
>
> # Calculation using pwr package
> pwr.r.test(n,r,sig.level,power,alternative)

     approximate correlation power calculation (arctangh transformation)

              n = 54.19491
              r = 0.37
      sig.level = 0.05
          power = 0.8
    alternative = two.sided

>
> # Specific Calculation
> r <- abs(r)
> p.body <- quote({
+   ttt <- qt(sig.level/2, df = n - 2, lower = FALSE)
+   rc <- sqrt(ttt^2/(ttt^2 + n - 2))
+   zr <- atanh(r) + r/(2 * (n - 1))
+   zrc <- atanh(rc)
+   pnorm((zr - zrc) * sqrt(n - 3)) + pnorm((-zr - zrc) * sqrt(n - 3))})
>
> n <- uniroot(function(n) eval(p.body) - power, c(4 + 1e-10, 1e+09))$root
>
> METHOD <- "approximate correlation power calculation (arctangh transformation)"
> structure(list(n = n, r = r, sig.level = sig.level, power = power,
+                alternative = alternative, method = METHOD), class = "power.htest")

     approximate correlation power calculation (arctangh transformation)

              n = 54.19491
              r = 0.37
      sig.level = 0.05
          power = 0.8
    alternative = two.sided

>
```

Supplementary Table S1. Correlations between mean gait parameters and hand grip strength.

	Mean	(SD)	*p*-value	*r*	Effect size	Interpretation	
Gait speed, m/s	0.87	(0.31)	**0.0002**	0.48	0.48	Medium	a
Stride length, m/HT	0.64	(0.14)	**0.0030**	0.39	0.39	Medium	a
Cadence, steps/min	101.6	(15.9)	**0.0011**	0.43	0.43	Medium	a
Stride time, sec	1.26	(0.32)	**0.0020**	-0.41	0.41	Medium	b
Stance time, sec	0.92	(0.27)	**0.0012**	-0.43	0.43	Medium	b
Swing time, sec	0.35	(0.06)	0.2679	-0.15	0.15	Small	b
Percentage of stance phase, %	71.4	(3.1)	<**0.0001**	-0.51	0.51	Large	b
Foot pitch angle at toe-off, deg	-46.8	(9.1)	0.0625	-0.25	0.25	Small	a
Foot pitch angle at heel contact, deg	18.7	(7.7)	**0.0139**	0.33	0.33	Medium	a
Minimum foot pitch angle, deg	-49.2	(9.8)	**0.0274**	-0.30	0.30	Small	a
Maximum foot pitch angle, deg	20.4	(8.1)	**0.0089**	0.35	0.35	Medium	a
Range of foot pitch angle, deg	69.6	(16.2)	**0.0077**	0.36	0.36	Medium	a
Minimum foot pitch angle time, sec	0.90	(0.29)	**0.0009**	-0.43	0.43	Medium	b
Maximum foot pitch angle time, sec	1.23	(0.29)	**0.0030**	-0.39	0.39	Medium	b
Timing of minimum foot pitch angle, %	69.9	(4.1)	**0.0002**	-0.48	0.48	Medium	b
Timing of maximum foot pitch angle, %	97.6	(3.4)	0.5526	-0.08	0.08	Negligible	b
Walk ratio, mm/(steps/min)	5.3	(2.1)	0.0605	0.25	0.25	Small	b
Foot speed during the swing phase, m/s	2.50	(0.73)	**0.0008**	0.44	0.44	Medium	a

Note: Bold values indicate p < 0.05. HT: Height. a: Pearson's test. b: Spearman's test.

Supplementary Table S2. Correlations between the coefficients of variation of gait parameters and hand grip strength.

	Mean	(SD)	*p*-value	*r*	Effect size	Interpretation	
Gait speed, %	16.0	(24.9)	**0.0242**	-0.30	0.30	Medium	b
Stride length, %	17.0	(36.4)	0.1157	-0.21	0.21	Small	b
Cadence, %	8.0	(8.7)	**0.0325**	-0.29	0.29	Small	b
Stride time, %	16.3	(24.5)	**0.0406**	-0.28	0.28	Small	b
Stance time, %	21.0	(31.2)	0.0576	-0.26	0.26	Small	b
Swing time, %	8.2	(7.3)	**0.0183**	-0.32	0.32	Medium	b
Percentage of stance phase, %	3.5	(4.0)	**0.0314**	-0.29	0.29	Small	b
Foot pitch angle at toe-off, %	9.2	(6.1)	0.3038	-0.14	0.14	Small	b
Foot pitch angle at heel contact, %	46.8	(129.4)	0.2036	-0.17	0.17	Small	b
Minimum foot pitch angle, %	11.2	(11.8)	0.3038	-0.14	0.14	Small	b
Maximum foot pitch angle, %	39.4	(66.8)	0.1105	-0.22	0.22	Small	b
Range of foot pitch angle, %	14.3	(17.4)	0.1265	-0.21	0.21	Small	b
Minimum foot pitch angle time, %	21.7	(31.0)	0.0557	-0.26	0.26	Small	b
Maximum foot pitch angle time, %	16.2	(24.2)	0.0562	-0.26	0.26	Small	b
Timing of minimum foot pitch angle, %	6.0	(11.5)	0.1385	-0.20	0.20	Small	b
Timing of maximum foot pitch angle, %	4.0	(7.3)	0.8956	-0.02	0.02	Negligible	b
Walk ratio, %	32.5	(72.6)	0.0989	-0.22	0.22	Small	b
Foot speed during the swing phase, %	10.4	(9.3)	0.0515	-0.26	0.26	Small	b

Note: Bold values indicate $p < 0.05$. a: Pearson's test. b: Spearman's test.
